# Evaluation of a Novel Assay for Detection of the Fetal Marker *RASSF1A*: Facilitating Improved Diagnostic Reliability of Noninvasive Prenatal Diagnosis

**DOI:** 10.1371/journal.pone.0045073

**Published:** 2012-09-14

**Authors:** Helen E. White, Carolyn L. Dent, Victoria J. Hall, John A. Crolla, Lyn S. Chitty

**Affiliations:** 1 National Genetics Reference Laboratory (Wessex), Salisbury District Hospital, Salisbury, United Kingdom; 2 Faculty of Medicine, University of Southampton, Southampton, United Kingdom; 3 Wessex Regional Genetics Laboratory, Salisbury District Hospital, Salisbury, United Kingdom; 4 Clinical and Molecular Genetics Unit, University College London Institute of Child Health, London, United Kingdom; 5 Fetal Medicine Unit, University College London Hospitals NHS Foundation Trust, London, United Kingdom; VU University Medical Center, Netherlands

## Abstract

**Background:**

Analysis of cell free fetal (cff) DNA in maternal plasma is used routinely for non invasive prenatal diagnosis (NIPD) of fetal sex determination, fetal rhesus D status and some single gene disorders. True positive results rely on detection of the fetal target being analysed. No amplification of the target may be interpreted either as a true negative result or a false negative result due to the absence or very low levels of cffDNA. The hypermethylated *RASSF1A* promoter has been reported as a universal fetal marker to confirm the presence of cffDNA. Using methylation-sensitive restriction enzymes hypomethylated maternal sequences are digested leaving hypermethylated fetal sequences detectable. Complete digestion of maternal sequences is required to eliminate false positive results.

**Methods:**

cfDNA was extracted from maternal plasma (n = 90) and digested with methylation-sensitive and insensitive restriction enzymes. Analysis of *RASSF1A*, *SRY* and *DYS14* was performed by real-time PCR.

**Results:**

Hypermethylated *RASSF1A* was amplified for 79 samples (88%) indicating the presence of cffDNA. *SRY* real time PCR results and fetal sex at delivery were 100% accurate. Eleven samples (12%) had no detectable hypermethylated *RASSF1A* and 10 of these (91%) had gestational ages less than 7 weeks 2 days. Six of these samples were male at delivery, five had inconclusive results for *SRY* analysis and one sample had no amplifiable *SRY*.

**Conclusion:**

Use of this assay for the detection of hypermethylated *RASSF1A* as a universal fetal marker has the potential to improve the diagnostic reliability of NIPD for fetal sex determination and single gene disorders.

## Introduction

Traditionally prenatal diagnosis of fetal genetic status has depended on the use of invasive diagnostic tests, either amniocentesis or chorionic villus sampling (CVS), which carry a small but significant risk of miscarriage [1] and cannot be performed until 11 weeks of gestation. However the identification of cell free fetal (cff) DNA in maternal plasma [2] has offered an alternative non invasive source of fetal genetic material for prenatal diagnosis. cffDNA originates from the apoptotic syncytial trophoblasts of the placenta [3], can be detected from 5 weeks gestation [4] and is cleared rapidly from the maternal circulation following delivery [5].

Analysis of cffDNA in maternal plasma is now in routine clinical diagnostic use for non invasive prenatal diagnosis (NIPD) where the target fetal sequence is derived from the father or where the allele arises *de novo*. NIPD for fetal sex determination can be carried out at an early gestational age for pregnancies known to be at risk of a sex-linked genetic condition [6], where a male fetus would be at risk of inheriting the condition, or conditions such as congenital adrenal hyperplasia where early treatment with dexamethasone can reduce the virilisation of female fetuses [7]. NIPD is used for fetal Rhesus D genotyping in D-negative mothers [8] and for some single gene disorders such as achondroplasia and beta-thalassemia [9], [10], [11].

Currently, clinical applications of NIPD require the detection of the paternally inherited (or *de novo*) allele to make a definitive diagnosis. Absence of detection of the paternal target sequence is either indicative of a true negative result or could also be due the lack of amplification of the sequence due to low concentrations of circulating cffDNA or the complete absence of cffDNA in the sample. Amplification of a fetal specific marker that confirms the presence of cffDNA allows a negative result to be more accurately interpreted as either a true or false negative result. The use of several fetal identifiers has been reported including the hypermethylated *RASSF1A* promoter [12], Y chromosome sequences for male pregnancies and panels of common polymorphic short tandem repeats, SNPs or indel markers [13], [14], [15], [16].

*RASSF1A* has been demonstrated to be hypermethylated in the placenta and hypomethylated in the maternal blood [12], [17]. Therefore, using methylation-sensitive restriction enzymes hypomethylated maternal sequences can be digested leaving only hypermethylated fetal sequences available for amplification by real time PCR. However, to eliminate the possibility of generating false positive results it is important to ensure the complete digestion of maternal hypomethylated sequences. Using previously published protocols we have detected up to 34% incomplete digestion of hypomethylated *RASSF1A* in clinical samples. Here we present a modified and simple real time PCR protocol that is applicable for the detection of hypermethylated *RASSF1A* promoter sequences in all pregnancies and demonstrate its clinical utility for fetal sex determination using *SRY* and *DYS14* real time PCR analysis.

## Materials and Methods

### Patient Samples

Informed consent for venepuncture was obtained from ninety pregnant women attending routine appointments with a community midwife (n = 62), at the early pregnancy unit (n = 4), at a routine ultrasound scan (n = 16) or for an invasive test procedure (n = 8) at Salisbury NHS Foundation Trust. The study was approved by the South West 1 Research Ethics Committee A (ref 09/H0104/59). Gestational age at blood collection was confirmed by routine ultrasound in all cases.

### Blood Collection and DNA Extraction

Maternal blood was collected into two 10 ml EDTA tubes and centrifuged at 1600 *g* for 10 minutes and the plasma fraction transferred to a 2 ml centrifuge tube and re-centrifuged at 20,000 *g* for 10 minutes. The cell free plasma fraction was stored at −80°C. Cell free DNA was extracted from 3 ml plasma using the Circulating Nucleic Acid Kit (Qiagen) following the manufacturer’s instructions and resuspended in 70 µl AVE buffer.

### Restriction Enzyme Digestion Real Time PCR

Restriction digestion reaction and real time PCR conditions were optimised on a separate training set of 90 plasma samples obtained from the SAFE-RAPID sample bank at Great Ormond Street Children’s Hospital prior to commencing this study [18]. Three 40 µl restriction enzyme digestion reactions were prepared for each sample: undigested control, methylation sensitive digestion and methylation insensitive digestion. Each reaction contained 20 µl cfDNA, 1× Buffer 4 (New England Biolabs) and no enzyme (undigested control), 2 U *TseI* and 4 U *BsmI* (methylation insensitive digest) and 2 U *BstYI* and 2 U *BstUI* (methylation sensitive digest). Samples were incubated at 60°C (undigested and methylation sensitive digest) and 65°C (methylation insensitive digest) for 2 hours. Additional restriction enzymes were then added to the reaction and the samples digested further at 37°C for 2 hours using no enzyme (undigested control), 8 U *EcoRI*, 8 U *MspI* and 4 U *HaeIII* (methylation insensitive digest) and 8 U *EcoRI*, 8 U *HhaI* and 4 U *HpaII* (methylation sensitive digest). All enzymes were supplied by New England Biolabs. cfDNA from male plasma was digested for each batch of samples analysed and performed as a digest control for the *RASSF1A* methylation insensitive and methylation sensitive digests. These digests should proceed to completion and therefore no *RASSF1A* amplification products should be observed following real time PCR. Further information regarding the selection of the restriction enzymes and details of the cleavage sites of the enzymes in the *RASSF1A* amplicon are given in Figure S3.

### Real Time PCR

5 µl of digested cfDNA was added to a 20 µl real time PCR containing 300 nM of forward and reverse primers for either *RASSF1A* or *SRY* (Table 1), 1X Power SYBR green PCR master mix (Life Technologies Corporation) and 0.2 U AmpErase Uracil N-glycosylase (Life Technologies Corporation). Products were amplified using a Rotorgene 6000 (Qiagen) with the following cycling conditions; 50°C for 10 minutes, 95°C for 10 minutes, 50 cycles of 95°C for 15 seconds, 60°C for 60 seconds acquiring on the Green channel. Samples were then melted from 65°C to 99°C with a 1°C increment at each step acquiring on the green channel. The threshold was set at 0.06 for all analyses.

**Table 1:** Sequences of PCR primers and probes for real time PCR assays.

Primer Name	Sequence 5′ to 3′	Reference
*RASSF1A* Forward	agcctgagctcattgagctg	[12]
*RASSF1A* Reverse	accagctgccgtgtgg	
*SRY* Forward	tggcgattaagtcaaattcgc	[4]
*SRY* Reverse	ccccctagtaccctgacaatgtatt	
*DYS14* Forward	gggccaatgttgtatccttctc	[19]
*DYS14* Reverse	gcccatcggtcacttacacttc	
*DYS14* Probe	FAM-6-tctagtggagaggtgctc–BHQ1	

Samples that were negative for *SRY* or had fewer than 9 positive replicates were also tested for the multicopy marker *DYS14* sequence of the *TSPY* gene on the Y chromosome [19]. 5 µl of digested DNA was added to a 20 µl real time PCR containing 300 nM of forward and reverse primers and 200 nM of probe (Table 1) and 1X TaqMan Universal PCR master mix (Life Technologies Corporation). Products were amplified using a Rotorgene 6000 (Qiagen) with the following cycling conditions; 50°C for 2 minutes, 95°C for 10 minutes, 50 cycles of 95°C for 15 seconds, 60°C for 60 seconds acquiring on the Green channel. The threshold was set at 0.06 for all analyses. No template controls and cfDNA extracted from a male plasma sample were included as negative and positive controls respectively for the *SRY*, *DYS14* and *RASSF1A* assays. cfDNA from male plasma acts as positive control for the amplification of *SRY* (all replicates), *DYS14* (all replicates) and *RASSF1A* (three replicates; undigested sample only). The cfDNA from male plasma also acts as a digest control for the *RASSF1A* methylation insensitive and methylation sensitive digests as digestion should proceed to completion and no *RASSF1A* amplification products should be observed.

### Data Analysis

**Figure 1:**
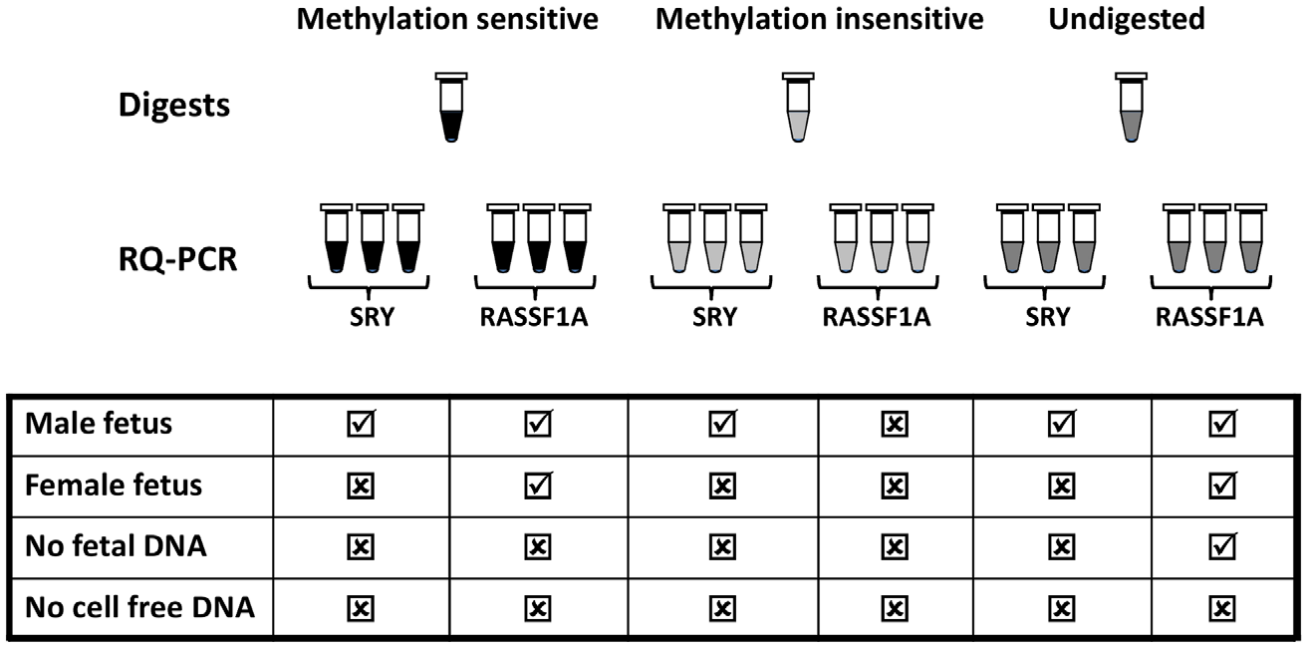
Schematic representation of the possible results obtained using hypermethylated *RASSF1A* as a universal fetal marker when determining fetal sex by NIPD for a male fetus, female fetus, a case where no cell free fetal DNA is present and a case where no total cell free DNA is present. Ticks represent the presence of amplified products from either the *SRY* assay or the *RASSF1A* assay. Crosses represent no amplification of *SRY* or *RASSF1A* amplicons.

Data analysis parameters were optimised on the training set of 90 plasma samples obtained from Great Ormond Street Children’s Hospital [18]. The presence of *RASSF1A* was determined by positive real time PCR amplification plots (Ct value <45) and a correct melt profile showing a specific peak at 91.2°C (Figure S1). Three replicates for each *RASSF1A* restriction digest were analysed. The no digest control (analyses total cfDNA) required 3 replicates to be positive for analysis to be undertaken. For the methylation insensitive digest (control for complete enzyme digestion of *RASSF1A* target sequence) complete digestion was assumed to have occurred if no replicates were positive. For the methylation sensitive digest, ≥2 out of 3 replicates were required to be positive to confirm the presence of hypermethylated fetal DNA. Samples with fewer than 2 positive replicates were not considered to have sufficient cffDNA present for reliable interpretation of the *SRY* result. Nine *SRY* (or *DYS14*) replicates were analysed for each patient sample (three from each digest) and the sample was considered to be male if 6 or more replicates showed amplification (Ct value <45) and a correct melt profile showing a specific peak at 80.5°C (Figure S1). Figure 1 shows the theoretical results for a male and female fetus, a case with no cffDNA, and a case with no cfDNA. All samples in this study were analysed in a blinded fashion.

## Results

### Sample Cohort Composition and Blood Transit Time

Fetal sex at delivery and pregnancy outcome were recorded for all samples. There were 37 female, 52 male and one case of female twins. Maternal ages ranged from 16–43 years (mean = 30 years) and gestational ages ranged from 5–23.2 weeks (mean = 9.1 weeks). All blood samples were received in the laboratory within 72 hours of collection.

### Real Time PCR Analysis for *RASSF1A* and *SRY*

All cases showed positive amplification of *RASSF1A* in the undigested sample indicating that total cell free DNA had been extracted successfully in all cases. Hypermethylated *RASSF1A*, was amplified in ≥2 out of 3 replicates for the methylation sensitive restriction enzyme digest for 79 samples (88%) indicating the presence of cffDNA. Of these, 45 showed amplification of *SRY* (≥6 out of 9 replicates positive) and were male at delivery and 33 had no detectable *SRY* (0 out of 9 replicates positive) and were female at delivery (including the XX twins). One sample was male at delivery but only showed amplification of *SRY* in only 3 out of 9 replicates and therefore this result would have been classified as inconclusive despite the detection of sufficient cffDNA. Hypermethylated *RASSF1A* was amplified in <2 replicates in 11 cases and these would have failed the data analysis parameters for reporting a clinical result. Of these, 10 samples had gestational ages less than 7 weeks 2 days which could explain the low level or absence of cffDNA in the samples [4]. Of the male samples (n = 6), four had fewer than 3 replicates positive for *SRY* (i.e. an inconclusive result) and two samples had 6 replicates positive for *SRY*. One male sample showed 0 replicates positive for both hypermethylated *RASSF1A* and *SRY* and therefore could have been mis-reported as female if the *RASSF1A* assay had not been used. All samples showed complete enzyme digestion of *RASSF1A* in the methylation insensitive digest reactions (see Figure S1 for examples).

### Real Time PCR Analysis of *DYS14* [19]

**Figure 2:**
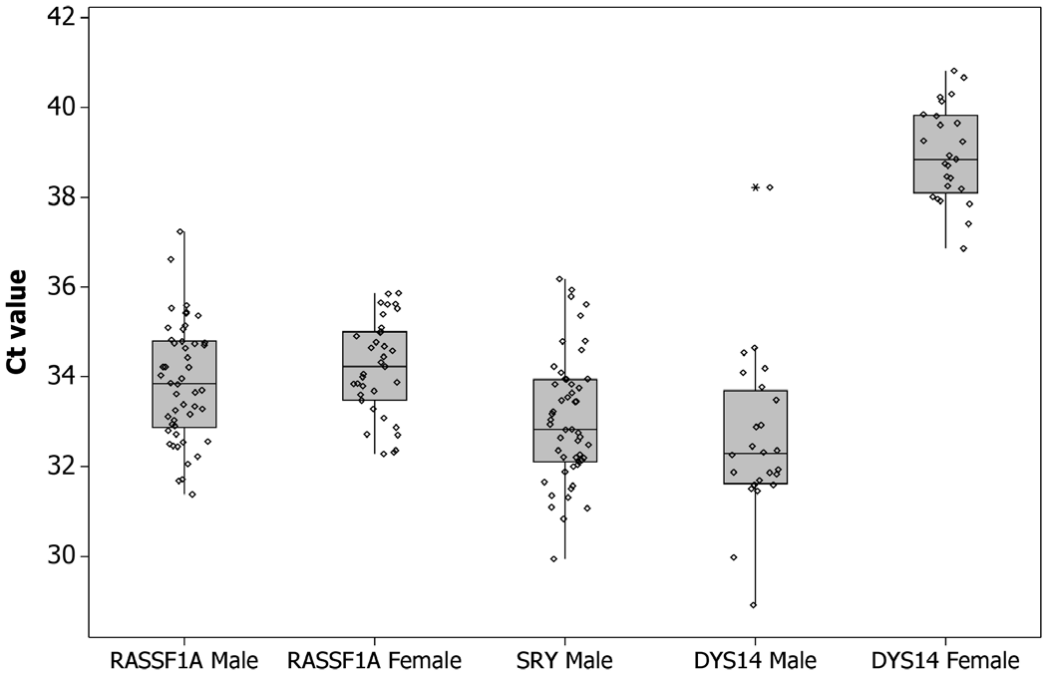
Box plots showing the Ct values for *RASSF1A*, *SRY* and *DYS14* real time PCR assays for male and female pregnancies. The mean Ct values were 33.9 (*RASSF1A*, male fetuses), 34.2 (*RASSF1A*, female fetuses), 33.0 (*SRY*, male fetuses), 0 (*SRY*, female fetuses), 32.5 (*DYS14*, male fetuses) and 39.2 (*DYS14*, female fetuses).

Samples that had fewer than 9/9 replicates positive for *SRY* were also tested for the multicopy marker sequence *DYS14* present in the *TSPY* gene on the Y chromosome (n = 61; 24 male, 37 female). Eleven female samples showed 0/9 replicates positive for *DYS14* but the remainder (n = 26) showed ≥1 replicate positive (range 1–8, mean 2.4) with an average Ct value of 39.2 (SD 1.7). The male samples (n = 24) had 9/9 *DYS14* replicates positive with an average Ct value of 32.5 (SD 1.8). The male sample that had no amplification of hypermethylated *RASSF1A* or *SRY* showed 9/9 replicates positive for *DYS14* however the mean Ct value was 38.2. Figure 2 shows box plots of the Ct values for *RASSF1A*, *SRY* and *DYS14* for male and female pregnancies.

## Discussion

Detection of hypermethylated *RASSF1A* promoter sequences can be used to confirm the presence of cffDNA in diagnostic samples. The *RASSF1A* promoter sequence is very GC rich and is therefore problematic to fully digest and amplify by real time PCR. We have made several modifications to previously published protocols [12], [20] to enhance the complete digestion of hypomethylated *RASSF1A* sequences, monitor that the digestion of the target has gone to completion and also improve the efficiency and interpretation of *RASSF1A* real time PCR. In preliminary experiments using published protocols we observed that incomplete digestion of *RASSF1A* was common in plasma samples derived from men and non-pregnant women where *RASSF1A* should not be observed following digestion with methylation sensitive enzymes (Figure S2a) and 25/73 (34%) of the samples from the initial training set where the Ct values of the *BstU1* only digested DNA [12] were lower than those digested with optimised digestion protocols (Figure S2b). Using our training set we undertook significant optimisation of the restriction enzyme digestion protocol with the addition of enzymes that cut outside the target amplicon to reduce genome complexity (Figure S3). This resulted in an improved protocol where the amplicon was shown to have been fully digested. Also, the low of efficiency of the hydrolysis probe assay (0.86, standard curve gradient −3.7) made interpretation of results difficult at high Ct values (low fetal DNA) as the amplification curves were not easily interpreted as being positive or negative. Therefore, we altered the real time PCR protocol to use SYBR Green rather than a hydrolysis probes as this resulted in improvements in the amplification efficiency to 0.98 (standard curve gradient −3.38). Use of a SYBR Green assay also enabled improvement in the interpretation of low level amplification signals by using melt curve analysis at the end of the real time PCR run. Inclusion of melt curve analysis allowed unambiguous interpretation of any non-specific amplification events that can occur when there are only low amounts of template DNA present. Only amplification events that resulted in a melt peak at 80.5°C or 91.2°C were scored as positive replicates for *SRY* and *RASSF1A* respectively (Figure S1). Although it may be desirable to multiplex the fetal target sequence and *RASSF1A*, using two differentially labelled hydrolysis probes, having a single SYBR Green *RASSF1A* assay means that this can be used in an economic manner in conjunction with other single gene tests e.g. rhesus D testing, achondroplasia without the need for extensive re-optimisation and validation of PCR multiplexes in a diagnostic setting.

Using the modified protocol we assessed the utility of the assay for confirmation of cffDNA when undertaking NIPD for fetal sex determination. Hypermethylated *RASSF1A* was amplified for 79 samples (88%) indicating the presence of cffDNA. For these samples the *SRY* real time PCR results and fetal sex at delivery were 100% concordant indicating that the test is 96–100% accurate with a 95% confidence interval [21]. As expected, insufficient levels of cffDNA (hypermethylated *RASSF1A*) were observed in most (n = 10) samples that had a gestational age of 7 weeks or less. This is concordant with a recently published study of a clinical audit of fetal sex determination that showed that discordant results could be obtained if testing is performed before 7 weeks [6]. Therefore, it is advisable that an accurate dating scan is performed prior to testing to ensure that the gestational age is >7 weeks. However, levels of cffDNA vary in pregnant women and the use of the *RASSF1A* assay should be considered for all samples to ensure that sufficient cffDNA is present to enable confident interpretation of a test result. For example, in this study two samples of gestational age <7 weeks had reportable levels of hypermethylated DNA and 7 replicates positive for *SRY* analysis and were phenotypically male at birth. Conversely a sample of gestational age 10 weeks 4 days showed unreportable levels of hypermethylated *RASSF1A* and an inconclusive number of positive *SRY* replicates. Using digital droplet PCR to quantify fetal load (*RASSF1A*) and fetal sex (*SRY*) it has been shown that the Pearson’s correlation coefficient between *SRY* and *RASSF1A* fetal loads is 97.3 with the *RASSF1A* fetal loads measured for some samples being lower than those determined using *SRY* [17]. It therefore appears that fetal DNA may not be completely hypermethylated in all samples. This may account for the cases which have an inconclusive *SRY* result but negative *RASSF1A* result**.** In all cases where unreportable levels of hypermethylated *RASSF1A* are obtained we recommend that repeat blood samples should be requested and testing repeated.

Analysis of samples for fetal sex determination using the multi copy marker *DYS14* has been reported as having a higher sensitivity of detection [19]. However in this study we observed that 70% of female samples showed at least 1/9 replicates positive for *DYS14* amplification although the Ct values for these samples (mean 39.2) were significantly higher than those obtained for male samples (mean Ct 32.5) (Figure 2). This has also been observed in an audit of reliability of NIPD for fetal sex determination where the same *DYS14* primer and probe set was used [16]. No amplification of *SRY* was observed in the same set of female samples and therefore, if only one Y chromosome target is used for analysis, we would recommend the use of *SRY* for fetal sex determination as there is less risk of reporting a false positive result.

This modified protocol for detection of fetal specific hypermethylated *RASSF1A* is applicable to all pregnancies since is it polymorphism independent and can be performed at the same time as the fetal sex determination on the same aliquot of cffDNA. The *SRY* and *RASSF1A* assays have identical reaction conditions and can be amplified in the same plate and therefore results can be reported within 48 hours of receipt of the maternal blood sample. The assay could also be used for NIPD of single gene disorders and fetal RhD status provided that the restriction enzymes do not cut within the target amplicon. Use of this assay for the detection of hypermethylated *RASSF1A* as a universal fetal marker has the potential to improve the diagnostic reliability of NIPD for fetal sex determination and single gene disorders.

## Supporting Information

Figure S1 Examples of PCR amplification plots and melting profiles for the *SRY* and *RASSF1A* real time SYBR green PCR assays.
(PDF)

Figure S2 **(a)** Amplification plots showing the difference in efficiency of three methylation sensitive *RASSF1A* restriction enzyme digest protocols using cell free DNA extracted from the plasma of a non-pregnant female. **(b)** Amplification plots showing an example of the difference in efficiency of two methylation sensitive *RASSF1A* restriction enzyme digest protocols using cell free DNA extracted from the same plasma sample from a pregnant female.
(PDF)

Figure S3 Details of restriction enzyme digests.
(PDF)
