# Supplementary material for: Evaluation of a Novel Assay for Detection of the Fetal Marker RASSF1A: Facilitating Improved Diagnostic Reliability of Noninvasive Prenatal Diagnosis
Source: PLoS One. 2012 Sep 14;7(9):e45073. doi: 10.1371/journal.pone.0045073 (PMC3443218; doi:10.1371/journal.pone.0045073)
Supplement: Figure S1 — Examples of PCR amplification plots and melting profiles for the SRY and RASSF1A real time SYBR green PCR assays. (PDF) [file pone.0045073.s001.pdf]

Figure S1: Examples of PCR amplification plots and melting profiles for the SRY and RASSF1A real time SYBR green PCR assays.

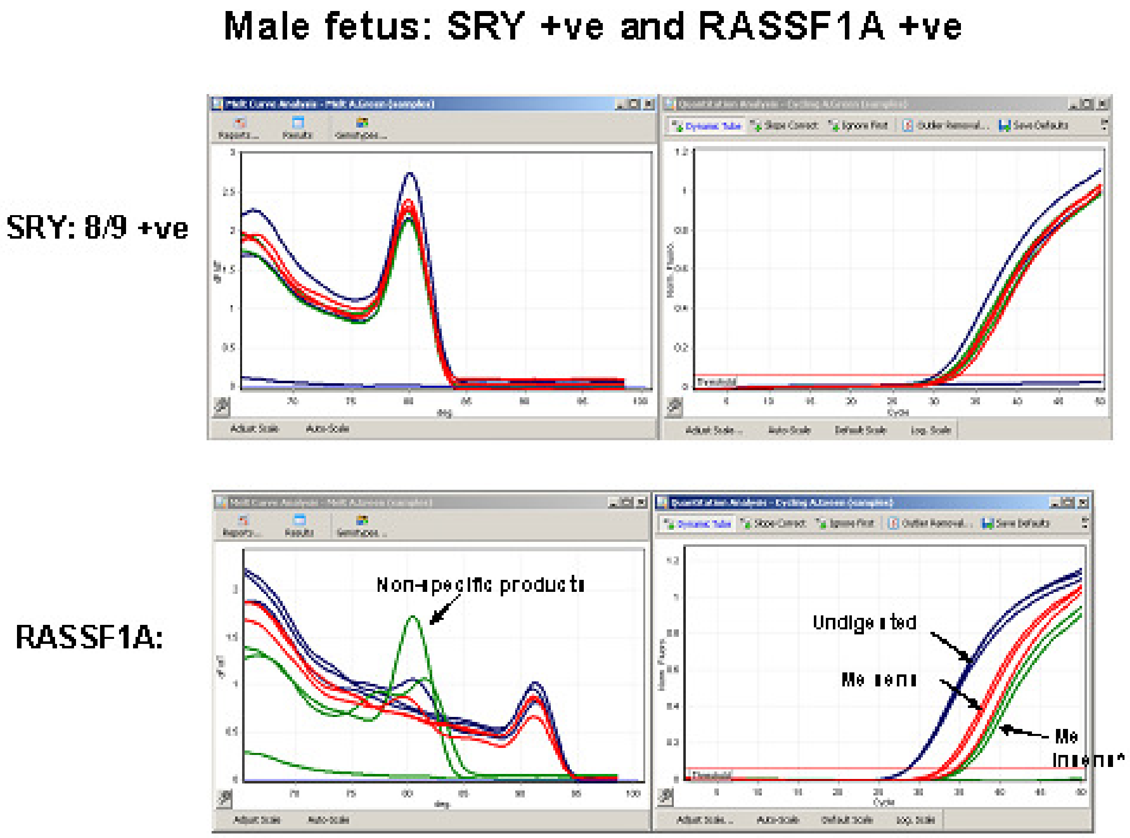

a) **Male fetus.** 8/9 amplification replicates were positive for *SRY* and melted at 80.5ºC indicating the presence of *SRY*. *RASSF1A* amplicons that melted at 91.2ºC were detected in the undigested control (blue lines) indicating that total cell free DNA has been extracted and the methylation sensitive digest (red lines) indicating the presence of cell free fetal DNA. The methylation insensitive digest failed to amplify RASSF1A products melting at 91.2ºC indicating complete digestion of cell free DNA fetus. Non specific amplification events were identified as positive amplification plots that failed to melt at the specified temperatures for SRY or RASSF1A. The fetus can be reported as male as 8/9 replicates were positive for SRY and the presence of cell free fetal DNA has been confirmed.

Female fetus: SRY -ve and RASSF1A +ve

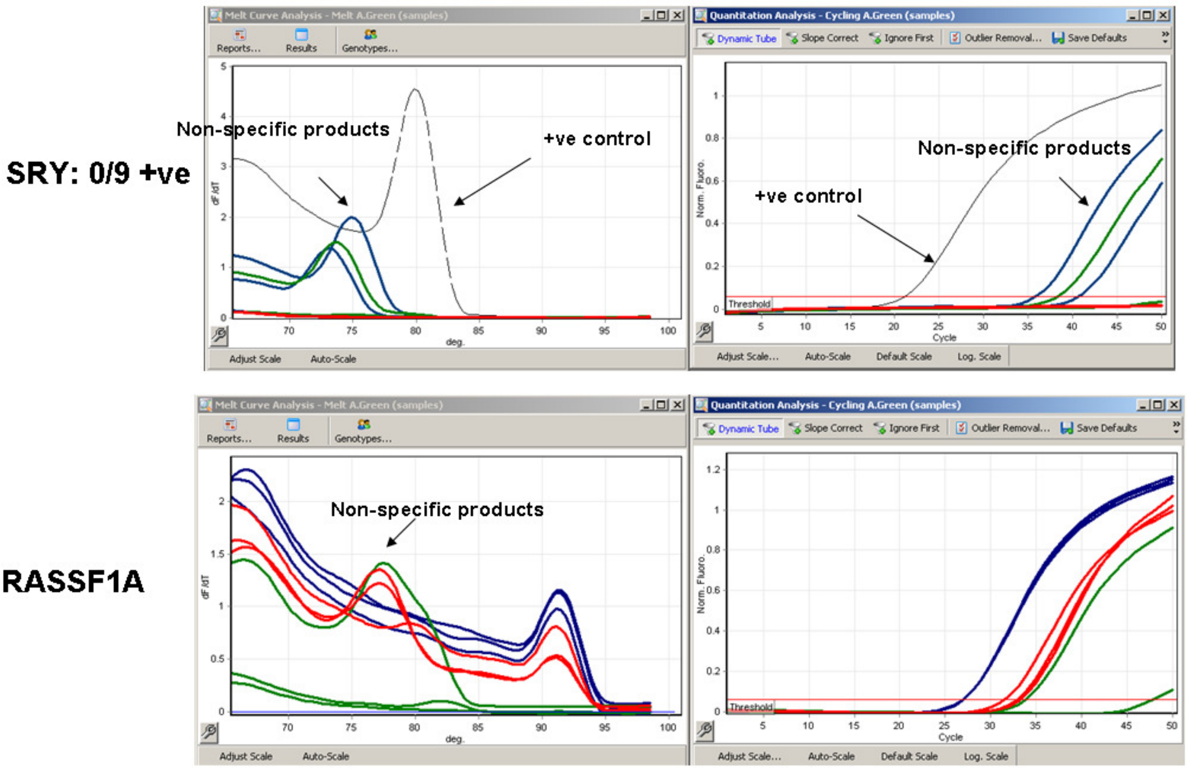

b) **Female fetus**. No *SRY* amplicons that melted at 80.5ºC were observed indicating absence of *SRY*. *RASSF1A* amplicons that melted at 91.2ºC were detected in the undigested control (blue lines) indicating that total cell free DNA had been extracted and in the methylation sensitive digest (red lines) indicating the presence of cell free fetal DNA. The methylation insensitive digest (green lines) failed to amplify RASSF1A products melting at 91.2ºC indicating complete digestion of cell free DNA. Non specific amplification events were identified as positive amplification plots that failed to melt at the specified temperatures for SRY or RASSF1A. The fetus can be reported as female as no SRY was detected and the presence of cell free fetal DNA has been confirmed.

No fetal DNA: SRY -ve and RASSF1A -ve

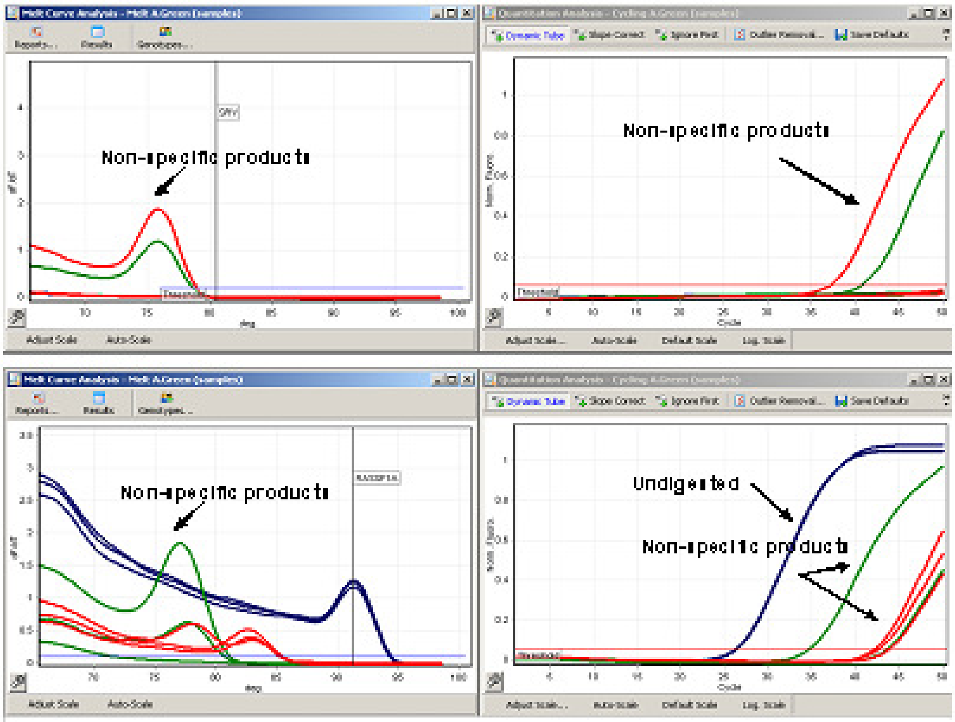

c) **No fetal DNA extracted**. No SRY amplicons that melted at 80.5ºC were observed indicating absence of SRY. RASSF1A amplifications that melted at 91.2ºC were detected in the undigested control (blue line) indicating that total cell free DNA has been extracted. However, no signal was detected in the methylation sensitive digest (red line) indicating the absence of fetal DNA. The methylation insensitive digest (green lines) failed to amplify RASSF1A products melting at 91.2ºC indicating complete digestion of cell free DNA. In this case the sample would fail analysis and a repeat sample should be requested. Without information from the RASSF1A assay this sample could have been reported incorrectly as a female.
