# Supplementary material for: Evaluation of a Novel Assay for Detection of the Fetal Marker RASSF1A: Facilitating Improved Diagnostic Reliability of Noninvasive Prenatal Diagnosis
Source: PLoS One. 2012 Sep 14;7(9):e45073. doi: 10.1371/journal.pone.0045073 (PMC3443218; doi:10.1371/journal.pone.0045073)

Figure S2a: Amplification plots showing the difference in efficiency of three methylation sensitive RASSF1A restriction enzyme digest protocols using cell free DNA extracted from the plasma of a non-pregnant female. The samples should digest completely and therefore no RASSF1A should be detected as there is no hypermethylated cell free fetal DNA present in the sample. Protocol D was the only one shown to give complete digestion.

A. Undigested samples (Ct 23.8)

B. Sample digested at 60°C with BstUI only (Ct 36.8) (e.g. ref 12).

C. Sample digested at 37°C with HpaII, HhaI and EcoRI (Ct 40.3).

D. Sample (purple line) digested at 60°C with BstUI and BstYI followed by digestion at 37°C with HpaII, HhaI and EcoRI. No amplification (purple line)

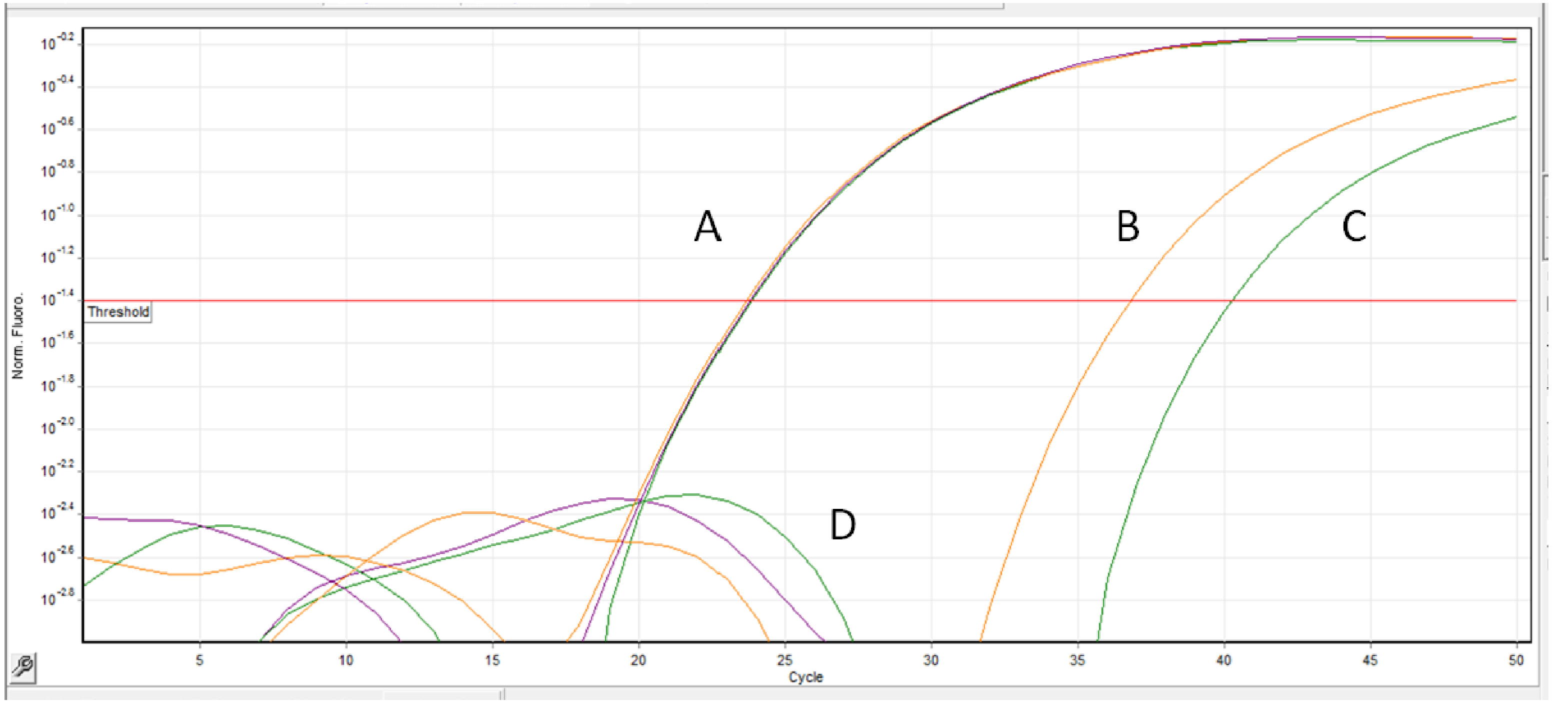

Figure S2b: Amplification plots showing an example of the difference in efficiency of two methylation sensitive RASSF1A restriction enzyme digest protocols using cell free DNA extracted from the same plasma sample from a pregnant female.

A. Undigested sample (Ct 29.1)

B. Plasma sample (red line) digested at 60°C with BstUI only (Ct 34.5)

C. Plasma sample (purple line) digested at 60°C with BstUI and BstYI followed by digestion at 37°C with HpaII, HhaI and EcoRI (Ct 35.6)

The difference in Ct values (1.1) between protocols B and C suggest that the digestion of unmethylated RASSF1A has been less efficient with protocol B than with protocol C. The amount of detectable RASSF1A is higher using protocol B than C an interpretation of that result is that protocol B leaves some of the unmethylated RASSF1A undigested.

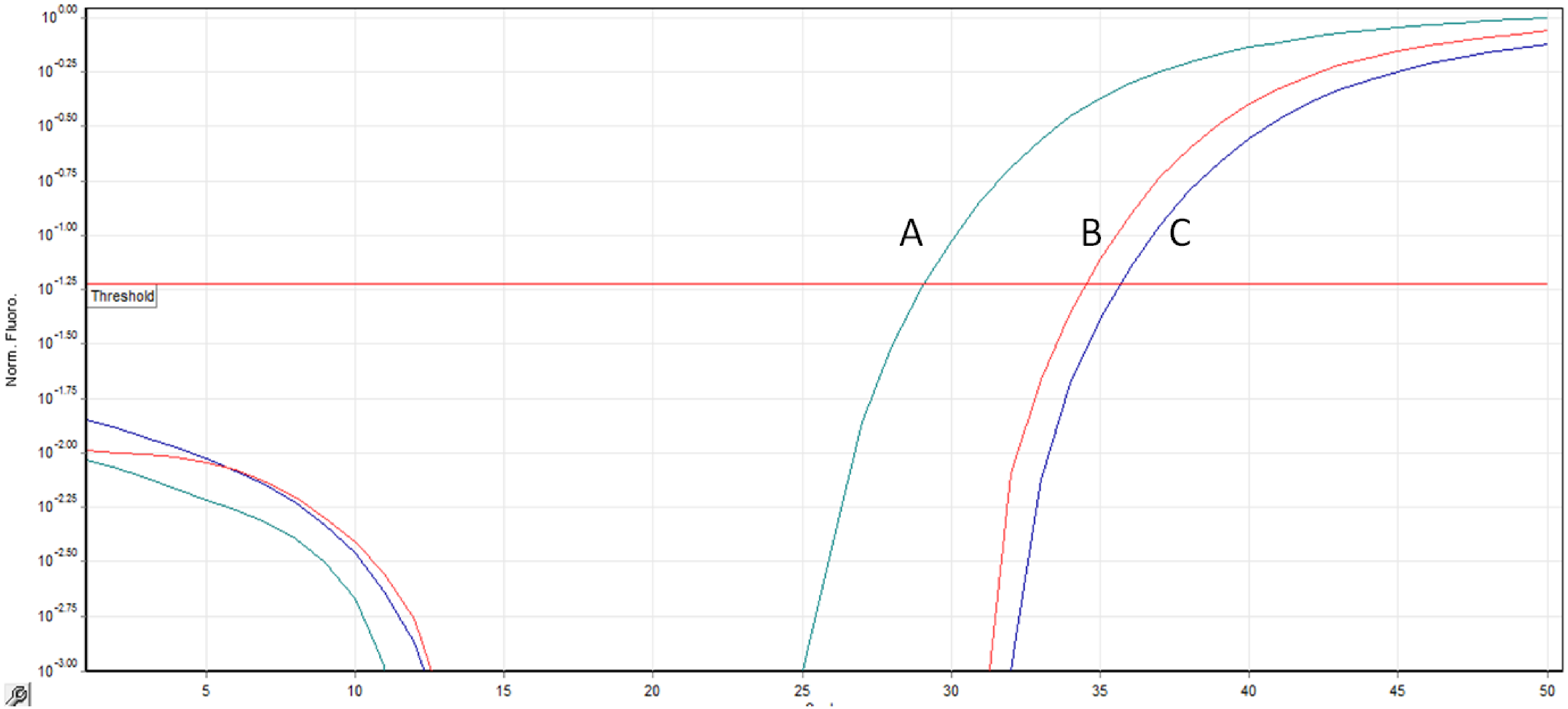

Supplement: Figure S2 — (a) Amplification plots showing the difference in efficiency of three methylation sensitive RASSF1A restriction enzyme digest protocols using cell free DNA extracted from the plasma of a non-pregnant female. (b) Amplification plots showing an example of the difference in efficiency of two methylation sensitive RASSF1A restriction enzyme digest protocols using cell free DNA extracted from the same plasma sample from a pregnant female. (PDF) [file pone.0045073.s002.pdf]
