# Supplementary material for: Evaluation of a Novel Assay for Detection of the Fetal Marker RASSF1A: Facilitating Improved Diagnostic Reliability of Noninvasive Prenatal Diagnosis
Source: PLoS One. 2012 Sep 14;7(9):e45073. doi: 10.1371/journal.pone.0045073 (PMC3443218; doi:10.1371/journal.pone.0045073)
Supplement: Figure S3 — Details of restriction enzyme digests. (PDF) [file pone.0045073.s003.pdf]

Supplementary figure 3: Details of restriction enzyme digests

a) Methylation sensitive digest of RASSF1A target amplicon: Enzymes will only cleave unmethylated DNA

agcctgagctcattgagctgcgggagctggcacccgctggg**cgcg**ctgggaagggccgcacccggctggagcgtgccaacgcgctgcgcatc**gcgcg**gggcac**cg**
cgtgcaaccccacacggcagctggt

Methylation sensitive enzymes cleave the amplicon at 11 sites:

60°C	**BstUI**	cleaves at CGCG	5 cuts at bases 43, 81, 93, 95, 105
37°C	HhaI	cleaves at GCGC	5 cuts at bases 43/41, 45/43, 83/81, 88/86, 95/93
	HpaII	cleaves at CCGG	1 cut at bases 62/64

EcoRI (37°C) and BstYI (60°C) are methylation sensitive and cut outside of the RASSF1A amplicon sequence and are used to reduce genome complexity

b) Methylation insensitive digest of RASSF1A target amplicon: Enzymes will cleave all DNA

Selecting a control for complete digestion of the unmethylated RASSF1A target can either be achieved by assessing the digestion of the target itself with enzymes that are insensitive to methylation or by digesting another target with the same enzymes. However, neither approach will prove that complete digestion of the unmethylated target has occurred, it will only reinforce the assumption that digestion has proceeded to completion.

Previously published protocols (e.g. 12) have used a surrogate unmethylated targets e.g. beta actin to infer complete digestion of the unmethylated RASSF1A target using the same enzymes. We elected to use a methylation insensitive digest to control for digestion of the target sequence itself as the RASSF1A amplicon sequence is 74% GC rich with a strong secondary structure. This requires the use of different enzymes to the methylation sensitive digest although isoschizomers were used where possible e.g. HpaII is an isoschizomer of MspI therefore cuts at the same recognition sequence but with differences only in methylation status. By using a control for the digestion of the target sequence itself (rather than a surrogate unmethylated target with different DNA sequence characteristics) we have used a methylation insensitive control that gives a more clear indication that the target itself was capable of digesting to completion in any given sample.

agcctgagctcattgagctgcgggagctggcacccgctgggcgcgctgggaag**ggcc**gcacccggctggagcgtgccaacgcgctgcgcatcgcgcggggcaccg
cgtgcaaccccacacggcagctggt

Methylation sensitive enzymes cleave the amplicon at **5** sites:

65°C	TseI	cleaves at GCWGC	3 cuts at bases 17/20, 83/86, 122/125
37°C	**HaeIII**	cleaves at GGCC	1 cut at base 55
	MspI	cleaves at CCGG	1 cut at base 62/64

EcoRI (37°C) and BsmI (65°C) cut outside of amplicon and are used to reduce genome complexity.

c) Digest controls

Cell free DNA extracted from a male plasma sample (contains no hypermethylated RASSF1A sequences) was included as a control in each batch of restriction enzymes digests to ensure that both digests proceeded to completion. Therefore no detectable signal for RASSF1A should be observed for these digests following real time PCR.

The SRY and DYS14 amplicons remain undigested throughout the procedure. There was no difference in the Ct values observed for either assay between the real time PCR reactions set up using template DNA from the undigested control (no enzymes added) and the methylation sensitive and insensitive digests. Therefore we conclude that there is no loss of fetal DNA during the digestion steps.

All enzymes were supplied by New England Biolabs and the digest reactions were performed in buffer 4 (New England Biolabs)
